# Transactions of the Chicago Society of Physicians and Surgeons

**Published:** 1875-01-01

**Authors:** Ralph E. Starkweather


### TRANSACTIONS OF THE CHICAGO SOCIETY OF PHYSICIANS AND SURGEONS.

### REGULAR MEETING, DECEMBER 14, 1874.

*Reported by Ralph E. Starkweather, M.D.*

DR. BARTLETT, President, in the chair. Dr. P. S. Hayes read a report of two cases of Amenorrhœa, which had been treated in the Woman's Hospital of the State of Illinois.

AMENORRHŒA.

The first case was one of amenorrhœa from infantile uterus and ovaries, and was treated by dilatation, local applications and electricity.

*History.*— Mrs. H., colored, twenty-three years of age, came under observation June 7th, 1873. Has always been of delicate constitution, and suffered from numerous ailments. Her first menstrual period occurred when she was seventeen years of age. Since that event, at irregular periods, there has been a slight sanguineous discharge from the vagina each month, she has had a return of pain in the back, limbs and head, accompanied occasionally by epistaxis — she is habitually constipated.

An examination made by Dr. A. Reeves Jackson, disclosed an infantile uterus. The sound passing into the uterus measured only three-fourths of an inch; neither ovary was distinguishable.

*Treatment.* — The uterus was dilated by means of the sponge tent which, however, gave rise to a slight peritonitis. The depth of the uterine cavity had been increased to one inch.

During the period of treatment, there occurred a sanguineous discharge from the vagina.

After an interval of five months, she resumed treatment, and Dr. Hayes employed the Faradic current, passing it transversely through the pelvis. Afterwards the galvanic current from fifteen elements was employed in conjunction with the Faradic. Improvement was only very slight, and then both currents were applied to the canal and cervix. After twenty-four applications of electricity, the uterus appeared to be in the same condition as at the commencement of treatment.

CASE II. Amenorrhœa, occurring after menstruation had been established, cured by the use of electricity.

Mrs. B., colored, twenty-seven years of age, began to menstruate at the age of fifteen, and was married at eighteen. Since her marriage, her health became much impaired, and her catamenia irregular both in respect to time and quantity. Three or four months frequently intervened between them. She has never been pregnant. She complains of an irritating leucorrhœal discharge and of pain in the head and in the mammary and ovarian regions, and of a frequent and painful desire to micturate — bowels constipated.

On examination by Dr. Jackson, it was found that the uterus was in its normal position, cervix a little elongated, the os was somewhat nodular, and had a tenacious mucous plug projecting from it. The sensitiveness of ths vaginal walls was abnormal. The breasts were long and flabby.

*Treatment* — A pill of aloes and myrrh, and electricity. Dr. P. S. Hayes applied the Faradic current, continued through ten minutes, as follows: One of the electrodes was placed alternately over each ovary and the uterus, the other electrode over either sacro-iliac synchondrosis, the current being frequently reversed, making the electrodes alternately positive and negative. The treatment was followed by a return of the catamenia, at first scanty and lasting for one day, the last two each continued three days and were quite normal in every respect; the disagreeable subjective symptoms had gradually disappeared. Twelve applications of electricity, similar to the one above described, had been given.

Dr. F. H. Davis read from advance sheets of THE MEDICAL EXAMINER a report of two cases under the care of Dr. N. S. Davis, of obscure nervous disease.

